# Changes of *KEAP1/NRF2* and *IKB*/NF-*κ*B Expression Levels Induced by Cell-Free DNA in Different Cell Types

**DOI:** 10.1155/2018/1052413

**Published:** 2018-03-20

**Authors:** Svetlana V. Kostyuk, Lev N. Porokhovnik, Elizaveta S. Ershova, Elena M. Malinovskaya, Marina S. Konkova, Larisa V. Kameneva, Olga A. Dolgikh, Vladimir P. Veiko, Vladimir M. Pisarev, Andrew V. Martynov, Vasilina A. Sergeeva, Andrew A. Kaliyanov, Anton D. Filev, Julia M. Chudakova, Margarita S. Abramova, Serguey I. Kutsev, Vera L. Izhevskaya, Nataliya N. Veiko

**Affiliations:** ^1^Research Centre for Medical Genetics (RCMG), Moscow 115478, Russia; ^2^V. A. Negovsky Research Institute of General Reanimatology, Federal Research and Clinical Center of Intensive Care Medicine and Rehabilitology, Moscow 107031, Russia; ^3^A. N. Bach Institute of Biochemistry, Research Center of Biotechnology, Russian Academy of Sciences, Moscow 119071, Russia; ^4^N. I. Pirogov Russian National Research Medical University, Moscow 117997, Russia

## Abstract

Cell-free DNA (cfDNA) is a circulating DNA of nuclear and mitochondrial origin mainly derived from dying cells. Recent studies have shown that cfDNA is a stress signaling DAMP (damage-associated molecular pattern) molecule. We report here that the expression profiles of cfDNA-induced factors NRF2 and NF-*κ*B are distinct depending on the target cell's type and the GC-content and oxidation rate of the cfDNA. Stem cells (MSC) have shown higher expression of *NRF2* without inflammation in response to cfDNA. In contrast, inflammatory response launched by NF-*κ*B was dominant in differentiated cells HUVEC, MCF7, and fibroblasts, with a possibility of transition to massive apoptosis. In each cell type examined, the response for oxidized cfDNA was more acute with higher peak intensity and faster resolution than that for nonoxidized cfDNA. GC-rich nonoxidized cfDNA evoked a weaker and prolonged response with proinflammatory component (NF-*κ*B) as predominant. The exploration of apoptosis rates after adding cfDNA showed that cfDNA with moderately increased GC-content and lightly oxidized DNA promoted cell survival in a hormetic manner. Novel potential therapeutic approaches are proposed, which depend on the current cfDNA content: either preconditioning with low doses of cfDNA before a planned adverse impact or eliminating (binding, etc.) cfDNA when its content has already become high.

## 1. Introduction

Cell-free DNA (cfDNA) is circulating DNA of both nuclear and mitochondrial origins. Dying cells are the major source of cfDNA [1–4]. For a long time, cfDNA has been studied as a passive marker of cell death after various influences, such as irradiation, and pathologies, especially oncologic [5, 6], or an object for noninvasive diagnostics (liquid biopsy), including prenatal [7–9]. Recently, a novel approach emerged to consider cfDNA as a signaling molecule, which is biologically active regardless of its nucleotide code sequence [1, 10, 11]. The signaling properties of cfDNA depend on two factors. First, it was shown that the GC-content of cfDNA differs from that of the source genomic DNA and depends on the pattern of cell death. In case of a chronic process, circulating cfDNA is enriched with CG-pairs due to the fact that GC-rich regions are more resistant to the endonuclease action [12, 13]. Second, cfDNA is prone to oxidation, mostly through the formation of 8-oxodG, and the oxidized cfDNA exerts a stronger signaling action in an oxidation degree-dependent manner [10, 14, 15].

The cfDNA is a DAMP (damage-associated molecular pattern) signaling molecule [16]. The DAMP signaling molecules are hypothesized to serve as messengers of infection or strongly hostile conditions/trauma provoking oxidative stress and cell death. The best-studied receptors for cfDNA are cytosolic AIM2, RIG-1, and DAI and some other DNA sensors [17–26], as well as TLR9 [27, 28]. It is commonly supposed that intrinsic DNA does not activate TLR9; however, our earlier studies have shown that TLR9 reacts for GC-rich endogenous cfDNA [29]. The activation of TLR9 evokes an inflammatory response that implicates the translocation of the transcription factor NF-*κ*B (nuclear factor kappa-light-chain-enhancer of activated B cells) from the cytoplasm to the nucleus with the subsequent launch of transcription of the NF-*κ*B-driven genes [30–33]. This is a tissue-level reaction.

At cellular level, an expression of 100+ genes providing for the cell protection in stress conditions is triggered by another transcription factor, NRF2 (nuclear factor- (erythroid-derived 2-) like factor 2) [34–36]. NRF2 is a master regulator of the antioxidative and anti-inflammatory cell responses [37–39] via the inducible expression of ARE- (antioxidant response element-) driven genes [40]. Thus, NRF2 can provide for the protection against stresses of chemical, infections, and other nature.

The interaction between NF-*κ*B and NRF2 is predominantly antagonistic [40–43]. The underpinning mechanisms are thoroughly reviewed in Discussion. At the same time, a number of stimuli such as reactive oxygen species (ROS), bacterial lipopolysaccharides (LPS), and oxidized low-density lipoproteins induce a simultaneous activation of both NRF2 and NF-*κ*B [44].

In case of protection failure at cellular level, the mechanism of programmed cell death is launched, because the evolutionarily formed strategy prefers to sacrifice the part for the benefit of the whole organism [45]. Both elevated and reduced cell death rates are deleterious and can entail certain pathologic conditions.

The proteins of the BCL2 family play a key role in the regulation of cell death and survival [46, 47]. The BCL-2 protein and four homologous proteins (Bcl-XL, Bcl-W, A1, and Mcl-1) favor cell survival [46].

The inhibitors of apoptosis proteins (IAP) repress caspases 3, 7, and 9 [48]. Under the stress conditions, when the cell survival-oriented processes are activated, expression of the antiapoptotic genes is induced [49].

The aim of this study was to explore the time dynamics of expression of the NF-*κ*B and NRF2 protective factors in response to the action of various kinds of cfDNA and in different cell types and to investigate the effect of cfDNA on cell survival and death.

## 2. Materials and Methods

Diverse aspects of the biological action of cfDNA were studied on histologically different cultivated cells with different proliferative capacity:

1. Mesenchymal stem cells (MSC) (*N* = 17) were derived from various sources and characterized by surface markers (Table 1) [15]: normal adipose tissue of surgical material after partial mastectomy (MSC AT), material of umbilical vein and umbilical blood (MSC V), and subcutaneous adipose tissue (MSC AT). The obtained profile of CD markers (Table 1) was typical for MSC [29].

The expression of surface proteins by the cells was studied with the help of flow cytofluorometry using the corresponding antibodies at CyFlow (PARTEC, Germany) [15].

(2) Cultures of human umbilical vein endothelial cells (HUVEC) (*N* = 9) were derived from 9 different specimens of umbilical vein (normal course of pregnancy, successful birth, and healthy newborns) [50]. The HUVEC were characterized by the CD31+ marker.
(3) Human breast adenocarcinoma cells (MCF7) were derived from the cell culture bank of Federal State Budgetary Institution “Research Centre for Medical Genetics” (RCMG), Moscow, Russia. The distinctive molecules of estrogen receptors (ER+) were located on the MCF7 surface [51].

### 2.1. Model cfDNA Fragment Samples

Based on the conclusions made from the results of our studies of cfDNA properties, we determined the most significant cfDNA parameters, which can evoke biological responses in different cell types:

1. Elevated GC-rich DNA content of the cfDNA, in particular, elevated ribosomal DNA (rDNA) content [14, 52].
2. Increased content of oxidized DNA fragments [15, 53].

In order to study the response to the presence of cfDNA in different cell types, model cfDNA fragments were used.

#### 2.1.1. Oxidized Forms of DNA

In case of pathologies and impacts deleterious for the genome, cfDNA contains an increased quantity of oxidized bases. Therefore, to investigate the action of oxidized DNA upon the cells of different types, we prepared *in vitro* samples of model oxidized forms of DNA (Table 2) [15]. We chose gDNA, which had been oxidized by Н_2_O_2_* in vitro*, as a model molecule in order to exclude the action of other possible factors, such as a changed methylation rate or shifted contents of various motifs that could exert influence on cfDNA properties.

The conditions of gDNA oxidation were chosen in such a way that the final content of the oxidation marker 8-oxo-deoxyguanosine in the oxidized gDNAoxy approximately corresponded to the real 8-oxo-deoxyguanosine content detected in the cfDNA in case of disorders accompanied by oxidative stress. Using mass spectrometry (ESI-MS/MS), we analyzed the 8-oxo-deoxyguanosine content in plasma cfDNA derived from patients with breast cancer and acute myocardial infarction and detected 800 and 2100 8-oxodG, respectively, per 10^6^ cfDNA nucleosides [15].

The specimens of oxidized DNA for the experiment were prepared using two methods: either treatment of a genomic DNA (gDNA) sample with 300 mM Н_2_O_2_/Fe^2+^/EDTA (gDNAoxy 1) or combined treatment of a gDNA sample with 300 mM Н_2_O_2_ and ultraviolet radiation at a wavelength of *λ* = 312 nanometers, which induced intense H_2_O_2_ decomposition and ROS production (gDNAoxy 2) [14]. The content of the oxidation marker 8-oxodG in the obtained DNA specimens was measured using mass spectrometry (ESI-MS/MS) (quantification of 8-oxodG was conducted by Galina V. Baidakova, a senior researcher of Federal State Budgetary Institution “Research Centre for Medical Genetics”) [15].

The 8-oxo-deoxyguanosine content in an intact gDNA was below the threshold sensitivity of the method, which was equal to 0.1 (8-oxodG)/10^6^ nucleosides, while the first gDNAoxy1 specimen contained ~400 (8-oxodG) per 10^6^ nucleosides (lightly oxidized DNA) and the second gDNAoxy2 specimen contained ~2900 (8-oxodG)/10^6^ nucleosides (highly oxidized DNA) [15].

When H_2_O_2_ is applied as an oxidizing agent, not only 8-oxodG but also some other oxidative modifications can be found in the DNA molecule after treatment, because H_2_O_2_ is a nonspecific oxidant. DNA can be oxidized with the formation of 8-oxodG only, if an oxidation technique based on methylene blue is used [54]. DNA oxidized in this way (DNA8-oxodG) contains solely 8-oxodG in a quantity of ~700 (8-oxodG)/10^6^ nucleosides, and we considered this a better model to explore the contribution of the 8-oxodG oxidative modification to the effects evoked by oxidized cfDNA *in vivo*. In addition, we oxidized the sequence of p(rDNA) in order to obtain GC-rich oxidized DNA with a very high oxidation rate of 50,000 per 10^6^ nucleosides [15].

#### 2.1.2. GC-Rich Model Fragments, Ligands for TLR9 and TLR9 Blockers

Earlier, we determined that in case of pathology, pregnancy, or damaging exposure, GC-rich rDNA fragments accumulate in the total pool of cfDNA, while the fraction of AT-rich satellite III (SatIII) fragments decreases. The corresponding model fragments were designed as plasmids containing rDNA or SatIII inserts.

A CpG-rich fragment of the transcribed region of the rDNA (from base pair 515 to 5321 in accordance with HSU13369, GeneBank) embedded in pBR322 vector (p(rDNA)) was used as the model GC-DNA. The GC-motif was 9504 bp long [55].

The model DNA samples underwent the same procedure of additional cleaning from lipopolysaccharides via a treatment with Triton Х-114 [52] with a subsequent gel filtration on the HW-85 carrier [55].

A computer-aided analysis of the nucleotide composition of p(rDNA) revealed unmethylated CpG motifs within rDNA, which are binding sites for TLR9, a TLR family receptor [55]. We conducted a thorough computer-aided analysis of the model plasmid samples used in the experiments for the existence of TLR9 binding sites and TLR9 blocking sequences.

The ligand for human TLR9 is the sequence GTCGTT and/or TCGTA [56–58]. Generally, R1R2CGY1Y2 is deemed to be an immunostimulating CрG-motif, where R1 stands for a purine (preferably G), R2 is a symbol for a purine or Т, and Y1 and Y2 are pyrimidines, which form a complex with human TLR9 having an association constant less than GTCGTT motif [57, 59]. TLR9 blockers can be the motifs Gn (*n* > 5), CCN(A/G/T)(A/G/T)NNGGGN, and CC(A/G/T)(A/G/T)NGGGNN [58, 60, 61].

The plasmid DNA we have chosen carries the pBR322 vector that harbors seven sites being the ligands for human TLR9. The plasmid p(rDNA) carries a CpG-rich fragment of the ribosomal repeat (which contains both binding sites and blocking sequences) [55].

### 2.2. Cultivation of MSC (Mesenchymal Stem Cells)

The technical problem of MSC cultivation is a requirement of the elimination of cells belonging to other tissues, which contaminate the MSC. If the selected cultivation conditions are optimum, the contaminant cells derived from other tissues are eliminated during subsequent passages [29]. The MSC were derived from the adipose tissue of the surgical material of patients with breast adenocarcinoma delivered from Federal State Budgetary Institution N. N. Blokhin Russian Cancer Research Center (Moscow) in one hour after partial mastectomy [29]. An informed consent for the use of the surgical material was obtained from each patient. The specimen was mechanically disintegrated in DMEM medium (PanEco, Moscow) containing gentamicin at 250 *μ*g/ml, penicillin at 60 units/ml, and streptomycin at 60 units/ml (PanEco); enzymatic dissociation was conducted in DMEM medium by incubating the preparation in the presence of 10% fetal bovine serum (PAA, Austria), 0.04% collagenase (Sigma), and the above-mentioned antibiotics for 16 h at 37°C [29]. The cells were centrifugated at 200*g* for 10 min, transferred into vials, and cultivated at 37°C in AmnioMax С-100 Basal Medium (Gibco) that contained AmnioMax Supplement C-100, 20 *μ*mol/l HEPES (PanEco) and the antibiotics [29].

### 2.3. Cultivation of HUVEC (Human Umbilical Vein Endothelial Cells)

Endothelial cells were isolated from the umbilical cord (healthy women, normal course of the pregnancy, birth in time and without complications, and healthy newborns). Material sampling and cell isolation were performed in sterile conditions pursuant to an adapted technique [62]. Cultivation was conducted in 199 medium (PanEco, Russia) with penicillin (50 units/ml), streptomycin (50 *μ*g/ml), gentamicin (10 *μ*g/ml), HEPES (20 *μ*l, PanEco, Russia), and growth factors at +37°C (starting density was 500,000 cells per 25 cm^2^).

### 2.4. Cultivation of MCF7 (Human Breast Adenocarcinoma Cells)

MCF7 were cultured in DMEM medium supplemented with 10% (*v*/*v*) fetal calf serum, 2 mM L-glutamine, 100 units/ml of penicillin, and 100 *μ*g/ml of streptomycin. Cells were grown in a humidified atmosphere with 5% CO_2_ in air at 37°C. Before treatment with DNA probes, cells were grown for 24 h or 72 h in slide flasks.

### 2.5. Measuring Gene Expression Levels Using Real-Time PCR

Expression levels of the genes *NFKB1*, *NRF2*, *BAX*, *BCL2*, *BCL2A1*, *BCL2L1* (*BCL-X*), *BIRC2* (*c-IAP1*), *BIRC3* (*c-IAP2*), *ТВР*, and *GAPDH* were measured using real-time PCR.

After the exposure of the cells to extracellular DNA fragments, RNA was extracted from the cells using YellowSolve kits (Clonogen, Russia) or Trizol reagent (Invitrogen) pursuant to the technique attached (http://tools.lifetechnologies.com/content/sfs/manuals/trizol_reagent.pdf) with the subsequent phenol-chloroform extraction and precipitation with chloroform and isoamyl alcohol (49 : 1). RNA concentrations were determined with the help of the dye Quant-iT RiboGreen RNA reagent (MoBiTec, Germany) at a plate reader (EnSpire equipment, Finland) (*λ*_excit_ = 487 nm, *λ*_flu_ = 524 nm). The reverse transcription reaction was carried out using chemical reagents supplied by Sileks company (Russia) according to the standard procedure.

PCR was carried out using the corresponding primers (Syntol) and the intercalating dye SybrGreen at StepOnePlus instrument (Applied Byosystems, USA). The used primers were as follows (written in the same order (F; R)): *NFKB1* (CAGATGGCCCATACCTTCAAAT; CGGAAACGAAATCCTCTCTGTT); *NRF2* (TCCAGTCAGAAACCAGTGGAT; GAATGTCTGCGCCAAAAGCTG); *ТВР* (5′-GCCCGAAACGCCGAATAT-3′; 5′-CCGTGGTTCGTGGCTCTCT-3′); *GAPDH* (GAAGGTGAAGGTCGGAGTC; GAAGATGGTGATGGGATTTC); *BAX* (CCCGAGAGGTCTTTTTCCGAG; CCAGCCCATGATGGTTCTGAT); *BCL2* (TTTGGAAATCCGACCACTAA; AAAGAAATGCAAGTGAATGA); *BCL2A1* (TACAGGCTGGCTCAGGACTAT; CGCAACATTTTGTAGCACTCTG); *BCL2L1* (CGACGAGTTTGAACTGCGGTA; GGGATGTCAGGTCACTGAATG); *BIRC2* (GAATCTGGTTTCAGCTAGTCTGG; GGTGGGAGATAATGAATGTGCAA); and *BIRC3* (AAGCTACCTCTCAGCCTACTTT; CCACTGTTTTCTGTACCCGGA).

The composition of the PCR reaction mix in a volume of 25 *μ*l was the following: 2.5 *μ*l of PCR buffer (700 mM/l Tris-HCl, pH 8.6; 166 mM/l ammonia sulphate, 35 mM/l MgCl_2_), 2 *μ*l of 1.5 mM/l dNTP solution, and 1 *μ*l of 30 picomol/l solution of each primer and cDNA. The conditions of PCR were chosen individually for each primer pair. The standard conditions for most primers were the following: after denaturation (95°C, 4 min), 40 amplification cycles were conducted in the following mode: 94°C for 20 sec, 56 to 62°С for 30 sec, 72°С for 30 sec, and then, 72°С for 5 min. The PCR procedures were performed at StepOnePlus (Applied Biosystems, USA).

Gene expression levels were analyzed in a series of independent experiments on cells from different donors. Statistical processing of the results was performed using a calibrating curve taking into account the PCR efficiency; the standard error was 2%.

The expression levels of pro- and antiapoptotic genes of interest were normalized to the expression levels of the respective standard gene (TBP) in each cell line analyzed.

Flow cytometry was applied to measure the content of 8-oxo-deoxyguanosine in nuclear DNA using primary (Sc-66036, Santa Cruz, USA) and secondary (anti-mouse-FITC, SC-2010, Santa Cruz, USA) monoclonal antibodies, double-strand DNA break rate via the analysis of gamma focuses of the phosphorylated form of H2AХ histone using antibodies to H2AX histones (NB100-78356G, NovusBio, USA), and protein expression level using the corresponding monoclonal antibodies BCL2 (Sc-783), BRCA2 (NBP1-88361), NOX4 (SC-30141), NRF2 (ab194984), p53 (sc-126-f), and PCNA (ab2426) according to the common protocol: the exposed cells and control cells were collected from the underlayer, washed with 1% albumin solution in PBS, fixed with 3.7% formaldehyde for 10 min at 37°C, washed off, and permeabilized in 90% methanol at −20°C. Then the cell suspension was incubated with primary antibodies (1 *μ*g/ml) overnight at +4°C (1 *μ*g/ml in PBS in the presence of 1% albumin) and, if necessary, with secondary antibodies (anti-rabbit-FITC Sc-2012, Santa Cruz, USA) for 1 h at room temperature in the dark and assayed with a flow cytofluorometer (СyFlow, Partek, Germany).

### 2.6. Fluorescence Microscopy

Fluorescence microscopy was conducted using fluorescence microscope AxioImagerA2 (Carl Zeiss). The cultured cells were fixed with 3.7% formaldehyde for 20 min at +4°C and permeabilized with 0.1% Triton X-100 in PBS (phosphate-buffered saline) with subsequent washing and blocking with 1% albumin solution in PBS and incubated overnight with primary antibodies to NRF2 and р65 subunit of NF-*κ*B at +4°C (1 *μ*g/ml in PBS in the presence of 1% albumin) and then, after washing with PBS, incubated for 1 h with secondary antibodies (Santa Cruz, USA) at room temperature, washed off with PBS and, if required, stained with DAPI.

### 2.7. Image Analysis

Image processing software “Image 6” was developed in our laboratory and applied in order to measure the fluorescence intensity in and around the nuclei and to calculate the content as compared to controls (in arbitrary units (arb.un.)).

The data were verified with the use of the multifunction system for cell imaging and subsequent automatic processing of the obtained data CyTell (GE Healthcare).

### 2.8. Annexin V Binding Assays

Cells were detached and washed with PBS. 10,000–50,000 cells were collected by centrifugation, washed in binding buffer (BB) (140 mM NaCl, 4 mM KCl, 0.75 mM MgCl2, 10 mM CaCl_2_, and 10 mM HEPES), and resuspended in 100 *μ*l of BB.

Annexin V-FITC/propidium iodide staining solution (to 10 samples: 4 *μ*l Annexin V-FITC (ab14082), 10 *μ*l of PI (50 *μ*g/ml), and 90 *μ*l BB, mixed well) was prepared and immediately added to the samples, then incubated at room temperature for 15 min in the dark. After staining, cells were immediately analyzed by flow cytometry using CyFlow Space (Partec, Germany). Annexin V-FITC binding (Ex = 488 nm; Em = 530 nm) was analyzed using the FITC signal detector (FL1) and PI staining by the phycoerythrin emission signal detector (FL2).

### 2.9. Statistical Analysis

The statistical data analysis was conducted using MS Excel, Statistica 6.0, StatGraph software. The null hypotheses of the absence of the difference between the compared samples were tested with the Mann–Whitney *U* test. Samples were deemed to be distinct at *p* < 0.05.

### 2.10. Ethics

The study design was reviewed and approved by the Local Ethics Committee of RSMG (Research Centre for Medical Genetics) to meet the requirements of the Helsinki Declaration of 1975 as revised in 2013. An informed consent for the use of the surgical material had been obtained from each patient, from whom an anonymous cell culture was derived.

## 3. Results

### 3.1. Nuclear Translocation of NF-*κ*B and NRF2 after Exposure to cfDNA

**Figure 1:**
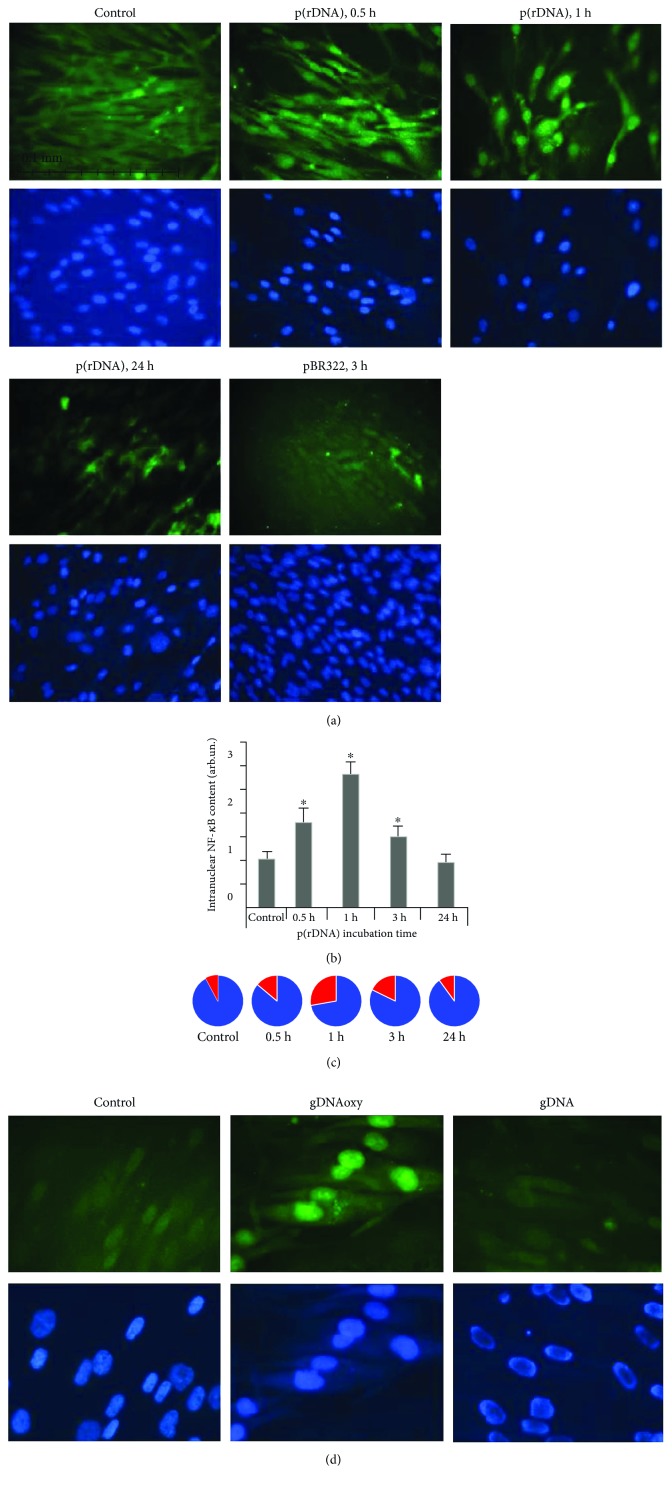
(a) Intracellular location of the р65 component of NF-*κ*B during cell incubation with GC-rich model DNA (p(rDNA)) and a plasmid vector carrying ribosomal repeats (pBR322) in a concentration of 50 ng/ml. The magnification was ×40; the exposure duration for NF-*κ*B is indicated in the figure. (b) Fraction of cells containing NF-*κ*B in the nucleus. The data were obtained using the Image 6 computer program for image analysis. The expression level of NF-*κ*B was calculated in relation to the control cells cultivated without adding cfDNA fragments. The control cells were taken as a unit. ^∗^*р* < 0.05. The experiment was conducted on two MSC cultures. For each culture, multiple measurements (three or more) were performed by different technicians. (c) Reads of the CyTell cell imaging system (GE Healthcare). The red sectors indicate the fraction of NF-*κ*B-positive nuclei, while the blue sectors indicate the fractions of NF-*κ*B-negative cell nuclei. The time of cultivation with p(rDNA) fragments and the vector (50 ng/ml) are indicated in the figure. (d) Intracellular location of NRF2 during cell incubation with oxidized cfDNA fragments (gDNAoxy) and genomic DNA (gDNA) in a concentration of 50 ng/ml. The exposure duration for NRF2 was 1 hour; the magnification was ×100.

The induction of the NF-*κ*B transcription factor by cfDNA fragments is followed by its nuclear translocation with the subsequent activation of target gene expression. The data for NF-*κ*B induction in MSC are shown in Figures 1(a) and 1(b). The translocation of the NRF2 factor under the action of oxidized cfDNA has a similar pattern (Figure 1(c)).

### 3.2. Profiles of NF-*κ*B and NRF2 Expression in Cells Exposed to cfDNA

**Figure 2:**
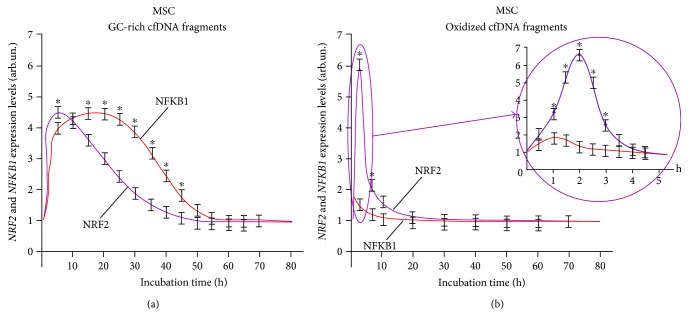
*NFKB1* and *NRF2* expression curves under the action of (a) GC-rich and (b) oxidized fragments in mesenchymal stem cells (MSC). The expression levels were determined every 5 minutes after adding the corresponding fragments (model GC-rich fragments, model oxidized fragments) using real-time PCR. For each gene, the expression level was calculated relating to the internal standard gene ТВР, with a spread in values at every point not exceeding 5%. ^∗^Significant difference (*p* < 0.05) between the relative values of NRF2 and NFKB1.

**Figure 3:**
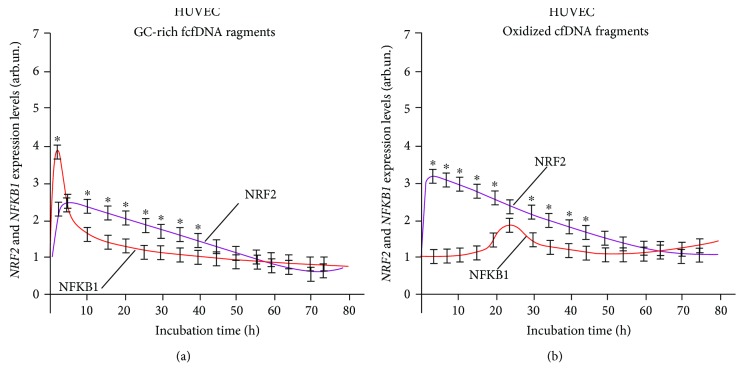
Interaction of two signaling pathways, NRF2 and NFKB1, in human umbilical vascular endothelial cells (HUVEC) exposed to (a) GC-rich and (b) oxidized cfDNA fragments. The expression levels were determined every 5 minutes after adding the corresponding fragments (model GC-rich fragments, model oxidized fragments) using real-time PCR. For each gene, the expression level was calculated relating to the internal standard gene ТВР, with a spread in values at every point not exceeding 5%. ^∗^Significant difference (*p* < 0.05) between the relative values of NRF2 and NFKB1.

**Figure 4:**
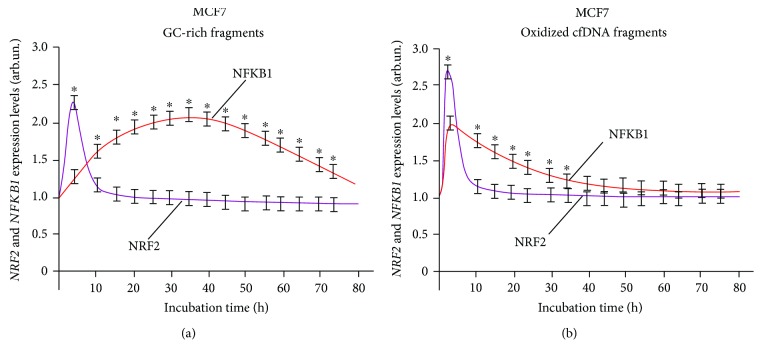
Interaction of two signaling pathways, NRF2 and NFKB1, in human breast adenocarcinoma cells (MCF7) exposed to (a) GC-rich and (b) oxidized cfDNA fragments. The expression levels were determined every 5 minutes after adding the corresponding fragments (model GC-rich fragments, model oxidized fragments) using real-time PCR. For each gene, the expression level was calculated relating to the internal standard gene ТВР, with a spread in values at every point not exceeding 5%. ^∗^Significant difference (*p* < 0.05) between the relative values of NRF2 and NFKB1.

Analysis of the dynamics of the expression of NRF2 and NF-*κ*B transcription factors (Figures 2, 3, and 4) corroborates the regularity of mutual inhibition of NF-*κ*B and NRF2 [40–43]. The expression of both factors starts almost simultaneously; however, the expression of NRF2 demonstrated a faster growth followed by NF-*κ*B suppression. Later, NF-*κ*B expression begins to increase, while NRF2 expression decreases. The expression profiles of the two factors overlap to a greater or lesser degree. The dynamics of these processes depends on the types of cells examined and cfDNA used for the induction of expression.

Interestingly, MSC demonstrated the early activation of transcription of *NFKB1* and *NRF2* genes in the presence of oxidized DNA fragments. However, the expression level of the *NFKB1* gene transcription factor only slightly increased in 30 minutes after the start of exposure and then the growth finished soon (in 1 hour), whereas the level of *NRF2* transcription increased (Figure 2(b)).

After the exposure of MSC to GC-rich fragments, the activation of *NFKB1* and *NRF2* gene transcription occurs later. The expression of *NFKB1* gene elevates in 3–24 hours. In contrast, transcription of *NRF2* begins to grow after 10 hours. In this case, we also observed a partial overlapping of the expression profiles of *NFKB1* and *NRF2* genes (Figure 2(a)).

After the exposure of human umbilical vascular endothelial cells (HUVEC) to GC-rich and oxidized fragments, the level of *NRF2* gene increased rapidly in 2-3 hours (Figure 3).

*NFKB1* gene transcription during exposure to GC-rich fragments was activated by 3 hours, with the expression level of this gene being approximately twofold higher than the level of transcription of *NRF2* gene—in this case, expression profiles of the two transcription factors under examination markedly overlapped (Figure 3(a)). Under the action of oxidized fragments, the level of *NFKB1* gene transcription peaked much later after 24 hours at the stage of the decreased transcriptional activity of *NRF2* gene (Figure 3(b)).

The human breast adenocarcinoma cells (MCF7) exposed to fragments of oxidized cfDNA and nonoxidized DNA showed a short-time increase in *NRF2* gene expression with the maximum level by 2 hours; then the content of RNA*NRF2* decreased (Figure 4).

Under the action of GC-rich fragments, a late elevation of *NFKB1* gene expression level occurred to have reached the maximum by 24 hours and persisted for a long time (Figure 4(a)). After the exposure to oxidized fragments, the maximum expression was registered in 3 hours, and partial overlapping of the *NFKB1* and *NRF2* gene expression profiles was observed (Figure 4(b)).

### 3.3. Apoptosis and Expression of Antiapoptotic Proteins under the Action of cfDNA

We observed no signs of massive cell death via necrosis in the cell culture exposed to cfDNA. Therefore, we studied the process of programmed cell death via apoptosis, a long-duration process estimated by specific markers. One of the most frequently used markers of apoptosis intensity is annexin V [63].

**Figure 5:**
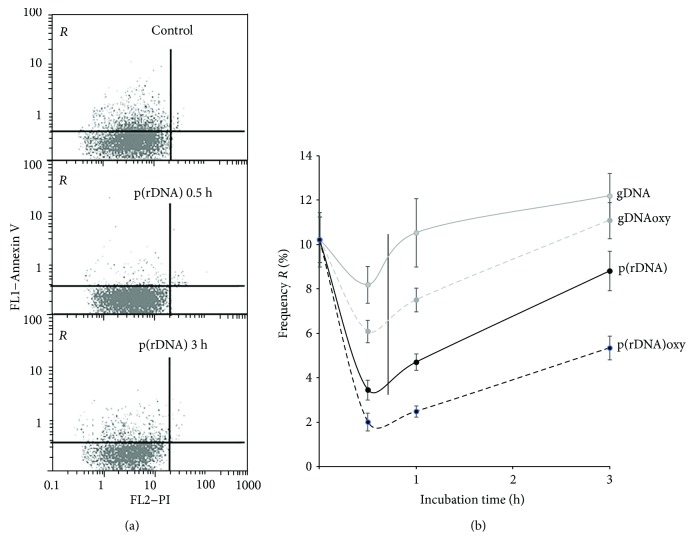
Time profiles of the fraction of apoptotic cells in HUVEC. (a) Annexin V fluorescence is plotted along the *y*-axis, and PI fluorescence is along the *x*-axis. From top to bottom: controls, after 1 hour and after 3 hours of exposure to p(rDNA) in a concentration of 50 ng/ml. (b) Temporal course of the frequency of apoptotic cells (cells belonging to the framed *R* fraction, i.e., with low PI and high annexin V fluorescence) in HUVEC culture according to the detection of cell surface levels of annexin V, a marker of early apoptosis during the cultivation with cfDNA fragments. Every curve significantly differs from the control (*p* < 0.05). gDNA: genomic DNA; p(rDNA): ribosomal repeat on a plasmid; gDNAoxy: oxidized genomic DNA; p(rDNA)oxy: oxidized ribosomal repeat.

In 30 minutes after adding DNA fragments in the HUVEC culture medium, the fraction of cells with the signs of apoptosis (fraction *R* framed in Figure 5(a)) diminishes, and this effect is especially prominent in case of exposure to GC-rich DNA (Figure 5(b)). Nonetheless, 3 hours later, the fraction of apoptotic cells increases after the exposure to any type of DNA.

**Figure 6:**
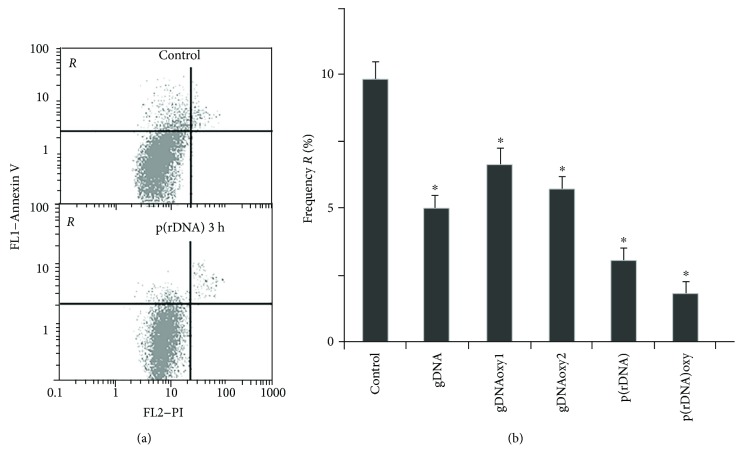
The exposure to cfDNA reduces apoptosis frequency in MSC. (a) Annexin V fluorescence is plotted along the *y*-axis, and PI fluorescence is along the *x*-axis. From top to bottom: controls, after 3 hours of exposure to p(rDNA) in a concentration of 50 ng/ml. *R* area contains cells with low PI and high annexin V fluorescence, that is, apoptotic cells. (b) The percentage frequency of cells belonging to the framed *R* fraction, that is, with the signs of apoptosis. ^∗^Significantly different from the control (*p* < 0.05). gDNA: genomic DNA; gDNAoxy1 and gDNAoxy2: oxidized genomic DNA with two different levels of oxidation; p(rDNA): ribosomal repeat; p(rDNA)oxy: oxidized ribosomal repeat.

The fraction of MSC with the signs of apoptosis after the exposure to nonoxidized, GC-rich, and oxidized cfDNA fragments was also estimated by the detection of annexin V protein on the cell surface (Figure 6).

A combination of oxidized and nonoxidized cfDNA fragments reduced the level of apoptosis in MSC registered in 3 hours by 40–50%. After a three-hour-long exposure, GC-rich oxidized and nonoxidized fragments (p(rDNA) and p(rDNA)oxy) caused a decrease in the frequency of cells with the signs of apoptosis in a greater degree by 70–80%.

A decrease in the fraction of dying cells in the cultures exposed to GC-rich and oxidized DNA is proved by a decrease several times in the content of endogenous extracellular DNA in the cell culture medium. During cultivation, endogenous DNA can be normally found in the medium. The contents are 23 ± 6 ng/ml in HUVEC cultures (a mean for three different cultures), 6 ± 5 ng/ml in MSC lines (a mean for six different cultures), and 140 ± 20 ng/ml in MCF7 cultures. This kind of cfDNA is derived from naturally dying cells during cultivation [16, 52].

Cells from a culture or from a body bind the endogenous extracellular/circulating DNA [64]. Cells seem to adapt to extracellular DNA in the medium and to be in an inactive state. We suppose that potential DNA binding sites, which are not blocked by endogenous DNA, remain on the cell surface in an inactive state. When the cfDNA content increases several times (it occurs *in vitro* after adding exogenous DNA to the culture medium or *in vivo* in case of massive cell death during acute pathologic processes), more DNA will bind to the cell surface. The process of cfDNA binding to the cell surface is rapid. Within the first 30 minutes after emerging the exogenous DNA in the medium, almost the entire amount of cfDNA is located on/in the cells, while the cfDNA content in the culture medium decreases below the control values. In the presence of oxidized (gDNAoxy, p(rDNA)oxy) and unoxidized GC-rich DNA (in a concentration of 50 to 100 ng/ml), the cfDNA content in the culture medium will decrease in 30 minutes by a factor of 2 and 1.5 (HUVEC, *N* = 3), 3.5 and 2 (MSC, *N* = 6), and 3 and 1.8 in the culture (MCF7) in relation to the baseline values measured before the cell exposure to the cfDNA fragments. We are of opinion that unoxidized cfDNA interacts with the cell surface during this process, while oxidized cfDNA is transported to the cytoplasm.

Fragments of GC-rich and oxidized cfDNA also reduced the strength of the apoptotic enzyme caspase 3 in HUVEC, MSC, and lymphocytes (*p* < 0.05). The influence of cfDNA on the strength of caspase 3 depends on the concentration and oxidation degree of the cfDNA fragments: low concentrations of oxidized cfDNA inhibited apoptosis in a greater degree than highly oxidized cfDNA. Fragments of GC-rich cfDNA inhibited apoptosis within a concentration interval of 5 to 100 ng/ml.

We studied the activation of the expression of genes for antiapoptotic proteins of the BCL-2 family (BCL2, BCL2A1, and BCL2L1), BIRC2 (c-IAP1), and BIRC3 (c-IAP2) after the exposure of different cell types to cfDNA.

**Figure 7:**
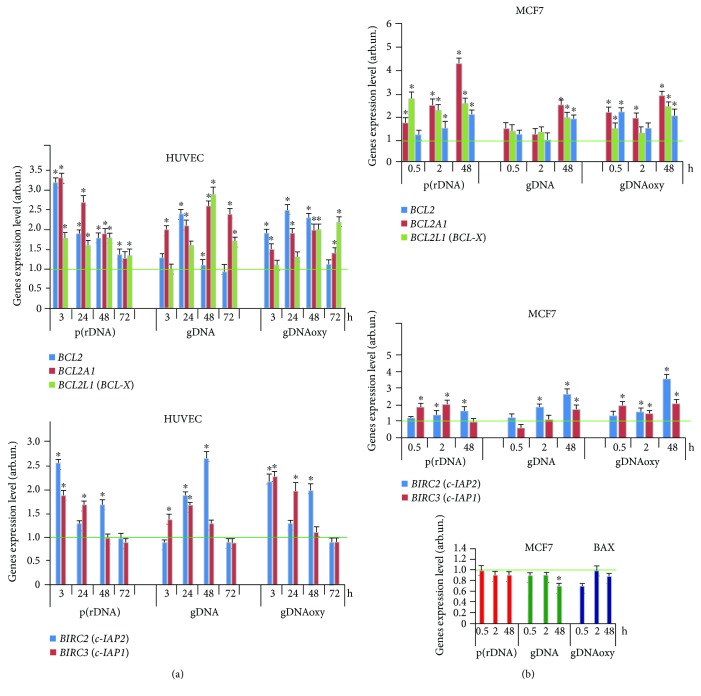
Expression levels of BCL2 family genes *BCL2*, *BCL2A1*, *BCL2L1*, *BIRC2* (*c-IAP1*), and *BIRC3* (*c-IAP2*) after an exposure of HUVEC (a) and MCF7 (b) to oxidized and nonoxidized GC-rich fragments of model cfDNA (50 ng/ml, see the exposure duration in the figure). Averaged values of three independent tests on HUVEC cultures derived from three different donors, and SD values are shown. As an internal standard gene, *ТВР* gene was used. The horizontal green lines show the mean gene expression level of intact endothelial cells (1 ± 0.2 arb.un.). ^∗^Values are significantly different from the control (*p* < 0.05, Mann–Whitney *U* test). gDNA: genomic DNA; gDNAoxy: oxidized genomic DNA; p(rDNA): ribosomal repeat.

The analysis of the amount of mRNA for BCL2, BCL2A1 (Bfl-1/A1), BCL2L1 (BCL-X), BIRC2 (c-IAP1), and BIRC3 (c-IAP2) in HUVEC showed that in response to an elevated cfDNA content, processes aimed to apoptosis prevention are considerably activated in the cells. This fact agrees with the data on the absence of significant changes of the total cell count, despite the proliferation arrest. The expression of BCL2 and BIRC family genes increases in 3 hours and remains elevated by a factor of 1.5 to 3 within 72 hours (Figure 7(a)). The activation of the antiapoptotic gene expression was also observed after the exposure of MCF7 culture to cfDNA.

**Figure 8:**
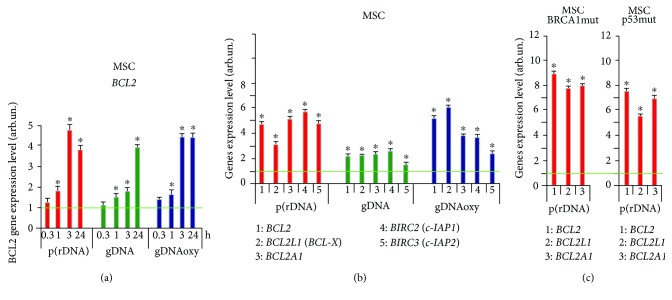
Expression level of *BCL2* gene (a) and *BCL2*, *BCL2A1*, *BCL2L1*, *BIRC2* (*c-IAP1*), and *BIRC3* (*c-IAP2*) genes (b) after an exposure of MSC to oxidized and nonoxidized GC-rich cfDNA fragments (50 ng/ml, see the exposure duration in the figure). Averaged values of three independent tests on MSC cultures derived from three different donors are shown. As an internal standard, ТВР gene was used. (c) Expression of *BCL2*, *BCL2A1*, and *BCL2L1* genes in MSC cultures with heterozygous mutations in *BRCA1* (5382insC) and *TP53* (L145p) genes. ^∗^Values are significantly different from the control (*p* < 0.05, Mann–Whitney *U* test). gDNA: genomic DNA; p(rDNA): ribosomal repeat.

In the presence of oxidized fragments (gDNAoxy and p(rDNA)oxy) and GC-rich fragments of p(rDNA) upon MCF7, the level of mRNA for *BCL2*, *BCL2A1*, and *BCL2L1* increases by a factor of 1.5 to 2 as early as in 0.5 hours, with a 2-fold to 4-fold increase by 48 hours (Figure 7(b)). Nonoxidized gDNA significantly (by a factor of 1.9 to 3.5) induced an increase in the expression of BCL2 family genes in MCF7 as late as 48 hours (Figure 7(b)). GC-rich and oxidized cfDNA fragments heightened *BIRC2* (*c-IAP1*) and *BIRC3* (*c-IAP2*) gene expression by a factor of 2 to 3 (Figure 8(b)). Under the action of cfDNA upon MCF7, the level of expression of the proapoptotic gene *BAX* does not increase or slightly decreases. The facts of the activation of the antiapoptotic genes and suppression of the proapoptotic gene *BAX* agree with the findings suggesting an augmentation of the fraction of the MCF7 pool under the exposure to cfDNA fragments. In MCF7, as well as in HUVEC, cfDNA blocks the process of apoptosis.

The expression of antiapoptotic genes also increases after an exposure of MSC to cfDNA. The expression of *ВCL2* gene increased in 1 hour by a factor of 1.5 to 2 on the average after adding oxidized and GC-rich cfDNA fragments; by 3 hours, the expression of *ВCL2* gene increased by a factor of 3.5 to 5 and remained on the same level in 24 hours (Figure 8(a)). The level of *BCL2*, *BCL2A1*, *BCL2L1*, *BIRC2* (*c-IAP1*), and *BIRC3* (*c-IAP2*) gene expression in MSC was heightened by a factor of 3 to 6 in 3 hours after the beginning of exposure of MSC to gDNAoxy and GC-rich p(rDNA); an exposure to gDNA increased the expression of the above-mentioned genes, on the average, two times only. Notably, MSC cultures that harbor mutations in *BRCA1* (5382insC) and *TP53* (missense mutation L145p in the fifth exon) genes showed more active expression of the antiapoptotic genes *BCL2*, *BCL2A1*, and *BCL2L1*: the level of expression of these genes raised approximately two times higher than in the MSC cultures carrying no mutation in *BRCA1* and *TP53* genes (data not shown). Apparently, such a strong antiapoptotic response in MSC cultures with mutations in *BRCA1* and *TP53* genes and the activation of the DNA repair gene *BRCA1* are aimed at the survival of cells with the defects in the genes of DNA repair and apoptosis regulation.

Highly oxidized DNA at a high concentration (300 ng/ml and higher) and high contents of p(rDNA) (>350 ng/ml) in the composition of cfDNA-induced cell death processes. So using Countess II FL Automated Cell Counter (TermoFisher) and cell staining with propidium iodide and annexin V-FITC, it was shown that the fraction of apoptotic cells in the MSC pool increased by 40% in 24 hours after the beginning of exposure to highly oxidized DNA in a high concentration (350 ng/ml and higher).

## 4. Discussion

Every complex metazoan organism maintains its homeostasis at several hierarchic levels: molecular, cellular, and tissue/organ. When the protection at a certain level is insufficient, the protection is activated at the higher level, while the lower level defense mechanisms are switched off in order to avoid excessive resource expenditures, and “broken” elements of the lower level are sacrificed in order to save the whole system. Similarly, the transition to the lower level of protection inactivates the higher level defense mechanisms and entails saving the lower level elements of the system. A good example of this “save-or-kill” strategy can be the well-investigated crosstalk between NRF2 (cellular level) and NF-*κ*B (tissue/organ level) defense pathways.

Most authors report the operation of the NF-*κ*B and NRF2 signaling pathways in opposition [41, 44, 65, 66]. The published articles propose a variety of mechanisms underpinning the mutual suppression of NRF2 and NF-*κ*B. Some of them are reviewed below.

Binding sites for NF-*κ*B were discovered in a rat *Nrf2* gene promoter [67]. NF-*κ*B binding to the *Nrf2* gene promoter is characterized by a feedback loop: after a long enough period of NF-*κ*B activity, the transcriptional activity of *Nrf2* increases thus suppressing NF-*κ*B [67].

KEAP1 was shown to have some homology to IKB. IKK*β* contains an ETGE motif [68]; therefore, it can bind KEAP1 and be targeted for ubiquitination [69]. Sequestration of the IKK*β* pool via KEAP1 binding reduces IKB*α* degradation and may be the elusive mechanism by which NRF2 activation is known to inhibit NF-*κ*B activation. When NRF2 is released due to oxidative signals, it results in an augmentation of the intracellular pool of unbound KEAP1, which can recruit more molecules of intracellular IKK*β* thus inhibiting the NF-*κ*B-driven gene expression. A mild oxidative stress entails a reversible KEAP1 alkylation; however, this reaction becomes irreversible under more oxidative conditions thus prohibiting most KEAP1 molecules from returning to the protein-binding conformation [70, 71]. In consequence of that, an abolishment of IKK*β* inhibition by KEAP1 is logically expected, which results in growing NF-*κ*B activation as more KEAP1 molecules lose their inhibitory properties. An additional argument in favor of this scheme is an experimentally established fact that the genetically determined decomposition of a KEAP1/CUL3/RBX1 complex with an E3-ubiquitin ligase that regulates both NRF2 and NF-*κ*B signaling pathways appeared to be the key mechanism triggering NF-*κ*B activation in human lung cancer cells [72]. KEAP1 has been shown to physically associate with NFKB-p65 *in vitro* and *in vivo*, and the signal from NF-*κ*B inhibits the NRF2 signaling pathway through the interaction between p65 and KEAP1 [73].

There is evidence for NRF2 modulating the NF-*κ*B signaling pathway at posttranslational level. This response involves IKB kinase and is mediated by the RAC1 signaling protein activated by the TLR4 receptor. This is a small GTPase of the RHO family, which is involved in innate immunity and triggers the NF-*κ*B signaling pathway, as well as activating the NRF2/ARE pathway, which in turn blocks the RAC1-dependent NF-*κ*B activation thus forming a negative feedback loop [74].

Seemingly, NF-*κ*B can repress the *NRF2* gene transcription by a mechanism connected with CREB: NF-*κ*B competes with NRF2 for a transcription coactivator CREB-binding protein (a protein that binds to CREB) [66].

**Figure 9:**
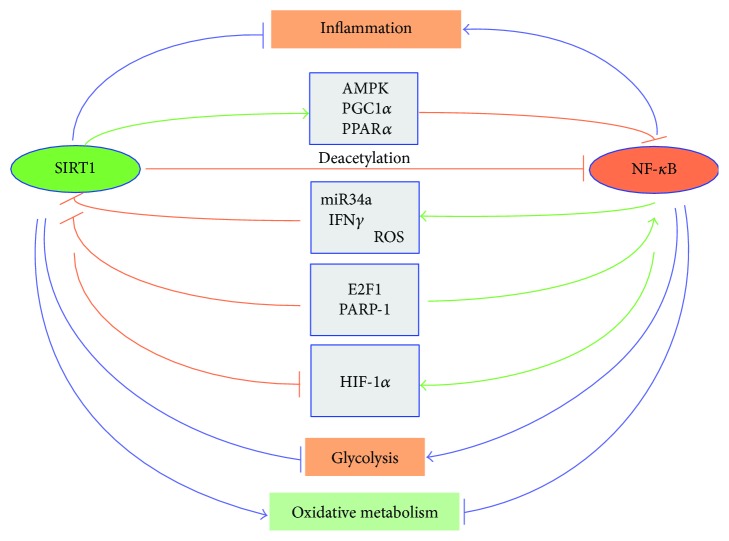
A schematic presentation of the antagonistic regulation between SIRT1 and NF-*κ*B signaling in the control of inflammation and metabolic responses. The major signaling pathways mediating this antagonistic regulation are shown.

Another scheme accounts for the antagonism because of an interaction of NF-*κ*B with histone deacetylase SIRT1 (Figure 9) [42]. In turn, SIRT1 is an upstream inductor of NRF2 [75–80].

Finally, direct sequestration of free radicals by the NRF2-driven enzymes weakens the action of NF-*κ*B [81].

Major NRF2 functions are xenobiotic detoxification and protection against oxidative stress. The oxidative stress is involved in the pathogenesis and progress of various diseases. Reactive oxygen species (ROS) alter the reductive-oxidative balance in the cells and apply oxidation-sensitive mechanisms in order to regulate the expression and activity of the transcription factors and genes regulated by the latter [82]. ROS trigger the NF-*κ*B signaling pathway resulting in elevated expression of a large quantity of proinflammatory cytokines such as TNF*α*, IL-1, IL-2, IL-6, IL-12, and adhesion molecules. The cytokines can induce ROS synthesis to form a vicious circle between oxidative stress and production of the proinflammatory cytokines [67]. This vicious circle can be broken by NRF2 released from the complex with its inactivator KEAP1 [65]. The activated free NRF2 translocates to the nucleus and launches the expression of genes for phase II detoxification enzymes and antioxidative enzymes, including NADP-H:quinone oxidoreductase 1 (NQO1), glutathione S-transferase (GST), heme oxygenase 1 (HO-1), glutathione peroxidase (GSH-Px), glutamate cysteine ligase (GCL), and peroxiredoxin 1 (PRX 1), which play an important role in cell protection by ROS quenching [67, 83]. The NRF2-KEAP1 system is acknowledged as the key mechanism of protection of the cell against oxidative stress. Besides, NRF2 inhibits the expression of proinflammatory cytokines, chemokines, adhesion molecules, matrix metalloproteinase (MMP-9), cyclooxygenase-2, and iNOS [67]. NRF2 modulates a cascade of anti-inflammatory cytokines via NF-*κ*B inhibition and regulates the antioxidant cellular responses [65]. Inducing NRF2 or inhibiting NOX4 as a source of ROS demonstrated therapeutic effectiveness *in vivo* in the therapy of diseases caused by cell senescence [84] or malignant transformation [85], via inhibiting NF-*κ*B.

**Figure 10:**
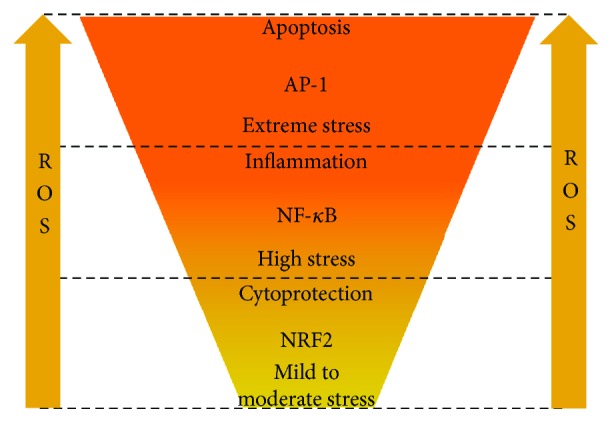
Differential responses to rising oxidative stress.

When the NF-*κ*B-mediated attempt to restore homeostasis fails and oxidative stress rises to extreme levels, AP-1-mediated apoptosis is triggered (Figure 10) [86, 87]. Thus, the organism successively implements the “save-or-kill” strategy firstly at the cellular level and, if it failed, then at the tissue level.

We studied the time profiles of activity of the transcription factors NF-*κ*B and NRF2 at the level of expression of their genes. Incubation with cfDNA was accompanied by an apparent increase in NRF2 expression in MSC and HUVEC at the levels of transcription and translation. The maximum effect was observed in case of action of oxidized DNA upon MSC, when the level of RNA*NRF2* showed a 12-fold increase and the protein level showed a 2-fold augmentation. In cancer cells, NRF2 plays an insignificant role in the response for a change of the parameters of cfDNA. The increased expression of NRF2 in MSC and HUVEC is followed by its nuclear translocation suggesting its activation as a transcription factor.

Our findings demonstrate a different intensity of the NRF2-based response in the cells of different types. We hypothesized that the organism's evolutionarily established readiness to sacrifice affected or damaged cells in order to save the tissue integrity depends on the degree of differentiation of the cells. Stem cells are more valuable as a cell depot; therefore, they showed the highest expression of *NRF2* in response to model cfDNA exposure. Perhaps, these peculiarities of MSC determine the anti-inflammatory effect in the MSC-based therapy of autoimmune diseases [88]. Unlike MSC, HUVEC are already differentiated cells; therefore, they easily develop an NF-*κ*B-mediated inflammatory response with the possible transition to massive apoptosis. The same picture was observed in other experiments on fibroblasts (data not shown). In the fibroblasts after serum withdrawal or an exposure to oxidized DNA, the levels of RNA*NRF2* and NRF2 protein increased fivefold.

As far as cancer cells are concerned, they are characterized by a secondary loss of specialization. The cancer cells are known to have constitutive activation of *NRF2* expression [89–93]; therefore MCF7 demonstrated the minimum response of *NRF2* expression to cfDNA, while the NF-*κ*B-mediated proinflammatory response prevailed.

The second regularity, which can be found in all the three cell type studies, consists in different responses for simple GC-rich cfDNA and for oxidized cfDNA. Probably, the evolutionarily established difference of these response patterns is caused by the fact that GC-rich cfDNA accumulates in the body during chronic cell death on a small scale. We showed earlier that under the above-mentioned circumstances, for example, in case of occupational exposure to low-dose ionizing radiation [13] or in case of a disease accompanied by elevated cell death [94], an activation of cfDNA-cleaving nucleases occurs. As a result, total blood cfDNA paradoxically decreases, while the fraction of GC-rich cfDNA with immunostimulatory properties increases. Oxidized cfDNA is a marker of the strong oxidative stress typical for acute conditions. Thus, GC-rich nonoxidized cfDNA is a signal of chronic mild cell death, while cfDNA with high 8-oxodG content is a hallmark of acute and massive cell death. We believe this difference underpins the observed diversity in the expression patterns. In every cell type studied, the response for oxidized cfDNA was more acute, that is, which started earlier and was more intensive, but returned faster to the baseline. GC-rich nonoxidized cfDNA evoked a weaker and elongated response with a predominance of the inflammatory component (NF-*κ*B). It is indeed quite reasonable that an acute oxidative stress requires a cytoprotective response, which can be provided by NRF2-driven genes, whereas the conditions of a chronic stress will transfer the response to the upper tissue level (inflammation).

The exploration of apoptosis rates after adding cfDNA showed that cfDNA with moderately increased GC-content and lightly oxidized DNA promoted cell survival.

The dose survival curves for various types of cfDNA (e.g., Figure 5(b)) are typical hormetic curves. Hormesis is a dose-response phenomenon characterized by a low-dose stimulation and a high-dose inhibition by the same signal [95]. Hence, the strategies of eventual modulation of the cfDNA effect for future therapeutic purposes should be different depending on the current cfDNA concentration: either preconditioning with low doses before a massive impact or measures intended to elimination (binding, etc.) of cfDNA in cases when the cfDNA concentration has become high.

In particular, the revealed effect of low cfDNA concentrations upon MSC suggests an alternative strategy to increase the viability of MSC used in transplantology and therapy of disorders followed by tissue degeneration, a short-time (3 to 24 hours) pretreatment (preconditioning) of MSC culture with a plasmid p(rDNA) in a low concentration (50 ng/ml). The benefits of the plasmid application are low active concentration and high resistance to nuclease cleavage.

Highly oxidized DNA at a high concentration (250 ng/ml and higher) and high contents of GC-rich DNA fraction in the composition of cfDNA induced apoptosis. We have supposed that the introduction of specific antibodies to cfDNA or blocking the cfDNA signal at the level of receptors will be able to neutralize the negative action of high cfDNA concentrations.

## Figures and Tables

**Table 1:** Surface marker profiles of MSC used in the study. Cell culture bank of Federal State Budgetary Institution “Research Centre for Medical Genetics.”

Number	Cells	Source	Surface markers
1	MSC AT (*N* = 9)	Breast adipose tissue	CD34−, CD45−, HLA-ABC+, HLA-DR−, CD44+, CD29+, CD49b low, CD54 low, CD90+, CD106−, CD105+, CD117−
2	MSC V (*N* = 5)	Umbilical blood and vein	CD34–, CD45–, HLA-ABC+, HLA-DR−, CD44+, CD29+, CD90+, CD105+, CD117−
3	MSC AD (hMADs) (*N* = 3)	Adipose tissue	CD34−, CD15−, HLA-ABC low, HLA-DR−, CD44+, CD13+, CD49b+, CD133−, CD90+, CD105+, CD117−

**Table 2:** Content of the oxidation marker 8-oxodG in DNA samples.

Number	Denomination	Content of 8-oxodG per 10^6^ DNA nucleosides
1	gDNA (control)	Less than 0.01
2	DNAoxy1	400
3	DNAoxy2 (or DNAoxy)	1400
4	DNA8-oxodG	700
5	p(rDNA)oxy	50,000
